# *Spag17* Deficiency Results in Skeletal Malformations and Bone Abnormalities

**DOI:** 10.1371/journal.pone.0125936

**Published:** 2015-05-27

**Authors:** Maria Eugenia Teves, Gobalakrishnan Sundaresan, David J. Cohen, Sharon L. Hyzy, Illya Kajan, Melissa Maczis, Zhibing Zhang, Richard M. Costanzo, Jamal Zweit, Zvi Schwartz, Barbara D. Boyan, Jerome F. Strauss

**Affiliations:** 1 Department of Obstetrics and Gynecology, Virginia Commonwealth University, Richmond, Virginia, United States of America; 2 Department of Radiology, Virginia Commonwealth University, Richmond, Virginia, United States of America; 3 Department of Biomedical Engineering, Virginia Commonwealth University, Richmond, Virginia, United States of America; 4 Department of Biochemistry and Molecular Biology, Virginia Commonwealth University, Richmond, Virginia, United States of America; 5 Department of Physiology and Biophysics, Virginia Commonwealth University, Richmond, Virginia, United States of America; Université de Lyon—Université Jean Monnet, FRANCE

## Abstract

Height is the result of many growth and development processes. Most of the genes associated with height are known to play a role in skeletal development. Single-nucleotide polymorphisms in the *SPAG17* gene have been associated with human height. However, it is not clear how this gene influences linear growth. Here we show that a targeted mutation in *Spag17* leads to skeletal malformations. Hind limb length in mutants was significantly shorter than in wild-type mice. Studies revealed differences in maturation of femur and tibia suggesting alterations in limb patterning. Morphometric studies showed increased bone formation evidenced by increased trabecular bone area and the ratio of bone area to total area, leading to reductions in the ratio of marrow area/total area in the femur. Micro-CTs and von Kossa staining demonstrated increased mineral in the femur. Moreover, osteocalcin and osterix were more highly expressed in mutant mice than in wild-type mice femurs. These data suggest that femur bone shortening may be due to premature ossification. On the other hand, tibias appear to be shorter due to a delay in cartilage and bone development. Morphometric studies showed reduction in growth plate and bone formation. These defects did not affect bone mineralization, although the volume of primary bone and levels of osteocalcin and osterix were higher. Other skeletal malformations were observed including fused sternebrae, reduced mineralization in the skull, medial and metacarpal phalanges. Primary cilia from chondrocytes, osteoblasts, and embryonic fibroblasts (MEFs) isolated from knockout mice were shorter and fewer cells had primary cilia in comparison to cells from wild-type mice. In addition, *Spag17* knockdown in wild-type MEFs by *Spag17* siRNA duplex reproduced the shorter primary cilia phenotype. Our findings disclosed unexpected functions for *Spag17* in the regulation of skeletal growth and mineralization, perhaps because of its role in primary cilia of chondrocytes and osteoblasts.

## Introduction

Human height is a highly heritable, classic polygenic trait [1]. Genetic alterations affecting height variation provide insight into human growth and development, as well as the genetic architecture of a number of diseases [2]. Prenatal longitudinal bone development and growth is a determinant of an individual’s postnatal height [3]. After formation of the cartilage anlage, longitudinal growth occurs at the epiphyseal growth plate, a layer of cartilage cells that are arranged in columns between epiphyseal and metaphyseal bone present in long bones and scapulae. In normal fetal growth plates, immature chondrocytes are located in a reserve cell zone (resting zone) that is oriented toward the epiphysis. Under hormonal, growth factor and mechanical regulation, these cells proliferate (proliferating cell zone), which increases length of the bone. After a set number of divisions, the cells undergo hypertrophy, remodeling their extracellular matrix to accommodate the change in cell shape (hypertrophic cell zone). Finally, the chondrocytes mineralize their extracellular matrix (calcifying cell zone), which is then vascularized, leading to bone formation [4]. In addition to long bone growth in the fetus, the diaphyseal bone also undergoes development with cortical bone forming around the cartilage anlage as the core is replaced with marrow [5,6].

Several genes have been shown to be involved in height [1,2,7–12], including those encoding proteins that are involved in signaling pathways known to be important in skeletal growth and development. Interestingly, single-nucleotide polymorphisms (SNPs) in the sperm associated antigen 17 protein gene (SPAG17) have been associated with human height [1,7,9–13] and idiopathic short stature in a Korean population [2]. However, it is not clear whether this gene directly influences height and/or skeletal growth.

*Spag17* encodes a protein present in cilia and flagella with a “9+2” axoneme structure. SPAG17 protein is present in the central pair complex (CPC). It is the orthologue of *Chlamydomonas reinhardtii* PF6 [14], a protein located on a projection from the C1 CPC microtubule in green algae [15]. Domains near the C-terminus of PF6 are essential for flagellar motility and assembly of the C1a projection. The N-terminal half is not required for the assembly, but it is important for stability of C1a complex members [16].

PF6 protein interacts with a number of other proteins, including calmodulin [17]. Consequently, PF6 is thought to be a proximal effector of CPC action on the sliding activity of the outer doublets that control cilia or flagellar beat. *Chlamydomonas pf6* mutants have paralyzed flagella, and lack the CPC 1a projection [15].

The murine *Spag17* gene encodes a full-length 250 kDa protein found in testis and tissues with motile cilia, but other splice variants and proteolytically generated fragments have been described [14,18]. Targeting of *Spag17* resulted in a severe phenotype characterized by motile cilia dysfunction in the nares and trachea, reduced clearance of nasal mucus, profound respiratory distress associated with lung fluid accumulation and disruption of the alveolar epithelium, cerebral ventricular expansion consistent with emerging hydrocephalus, failure to suckle, and neonatal demise within 12 h of birth. Ultrastructural analysis revealed loss of one CPC microtubule in approximately one quarter of tracheal cilia axonemes, absence of a C1 microtubule projection, as well as less frequent CPC structural abnormalities [19].

In the present study, the effect of a targeted mutation in *Spag17* on the skeleton during differentiation, maturation and growth was examined. The effect of silencing of *Spag17* ^*-/-*^ on osteoblasts and chondrocytes was examined to evaluate the role of *Spag17* in cells involved in osteochondral development. Our findings suggest that SPAG17 protein plays a role in endochondral bone formation, most likely because of a function in primary cilia.

## Results

### *Spag17* plays a role in skeletal development

*Spag17* ^*-/-*^ mice have a severe phenotype characterized by motile cilia dysfunction [19]. Here we show that *Spag17* ^*-/-*^ mice also have skeletal malformations. This finding was verified using a variety of experimental methods and provides evidence for a functional role for SPAG17 in height, consistent with multiple genetic association studies.

#### *Spag17*-deficient mice exhibit defects in development of the femur

**Fig 1:**
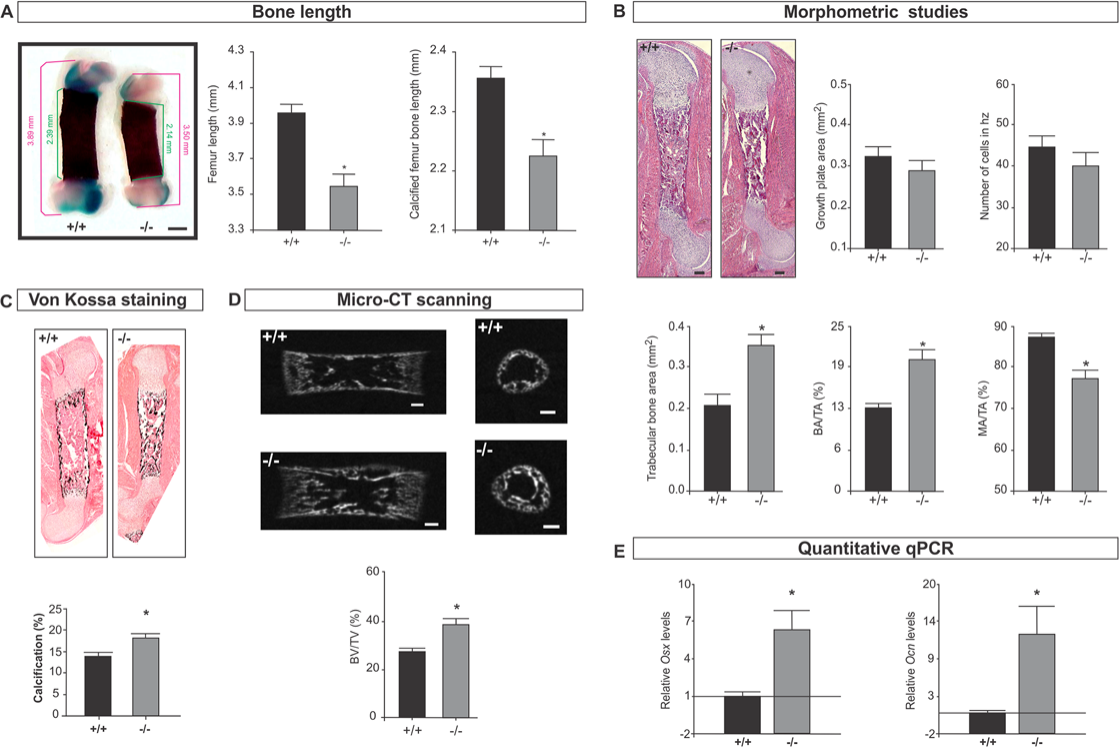
Femur bone development is affected in *Spag17*-deficient mice. (A) Alcian Blue/ Alizarin Red staining of femur from wild-type (+/+) and *Spag17*-mutant (-/-) mice. Measurement of femur length and calcified femur bone length shows *Spag17*-mutant limbs are shorter than wild-type mice. Scale bars, 0.5 mm. (B) Histological and morphometric studies on femur shows increase bone formation in mutant mice. Scale bars, 200 μm. (C) von Kossa staining reveals increased calcification in mutant mice. (D) Micro-CT structural analysis on femurs; mid-sagittal sections (left) and cross-sectional trans-axial views at the mid-diaphysis (right). Images were acquired in 600 projections in 180 degrees of rotation with 500 miliseconds exposure per projection. Scale bars, 200 μm. (E) mRNA expression of *osterix* (Osx) and *osteocalcin* (Ocn) measured by qPCR. (+/+), wild-type; (-/-), *Spag17*-mutant mice. Data are presented as means ± SEM from ≥7 mice. * Indicates statistically significant differences, p< 0.05. hz: hypertrophic zone; BA/TA: bone area/total area; MA/TA: marrow area/total area; BV/TV: bone volume/total volume; *Osx*: *osterix*; *Ocn*: *osteocalcin*.

Because SPAG17 has been associated with human height [1,7,9–13], we investigated hind limb length. Femurs and tibias from wild-type and mutant mice were isolated, stained with Alcian blue and Alizarin red for cartilage and bone detection, respectively, and then length was measured as described in Materials and Methods. Fig 1A shows that femurs from mutant mice are significantly shorter than wild-type mice. However, no differences in bone width were observed. Morphometric studies showed normal cartilage development without significant differences in the growth plate area and number of cells in the hypertrophic zone. Trabecular bone area and bone area/total area were increased, leading to a reduction in marrow area/total area in the mutant mice (Fig 1B and S1 Fig). Because bone formation is related to bone mineralization, we examined the mineral deposits by von Kossa staining. This analysis revealed significant differences indicating that the *Spag17* mutation has a significant effect on mineral deposition within the bone matrix (Fig 1C and S1 Fig). Increases in the ratio of BV/TV(%) were also observed by micro-CT scanning (Fig 1D). In addition, the expression of two genes involved in osteogenesis, *osterix* (*Osx*) and *osteocalcin* (*Ocn*), was higher in the mutant mice in comparison to wild-type mice (Fig 1E). Osteoclasts are cells that degrade bone to initiate normal bone remodeling and mediate bone loss in pathologic conditions and by increasing their resorptive activity. Although osteoclast numbers were slightly reduced in femurs of knockout mice, the reduction was not statistically significant, S3 Fig Together, the data collected from the femurs suggest that the *Spag17*-mutant mice have enhanced bone formation, which may lead to bone shortening due to premature ossification.

#### *Spag17-*deficient mice exhibit defects in tibia development

**Fig 2:**
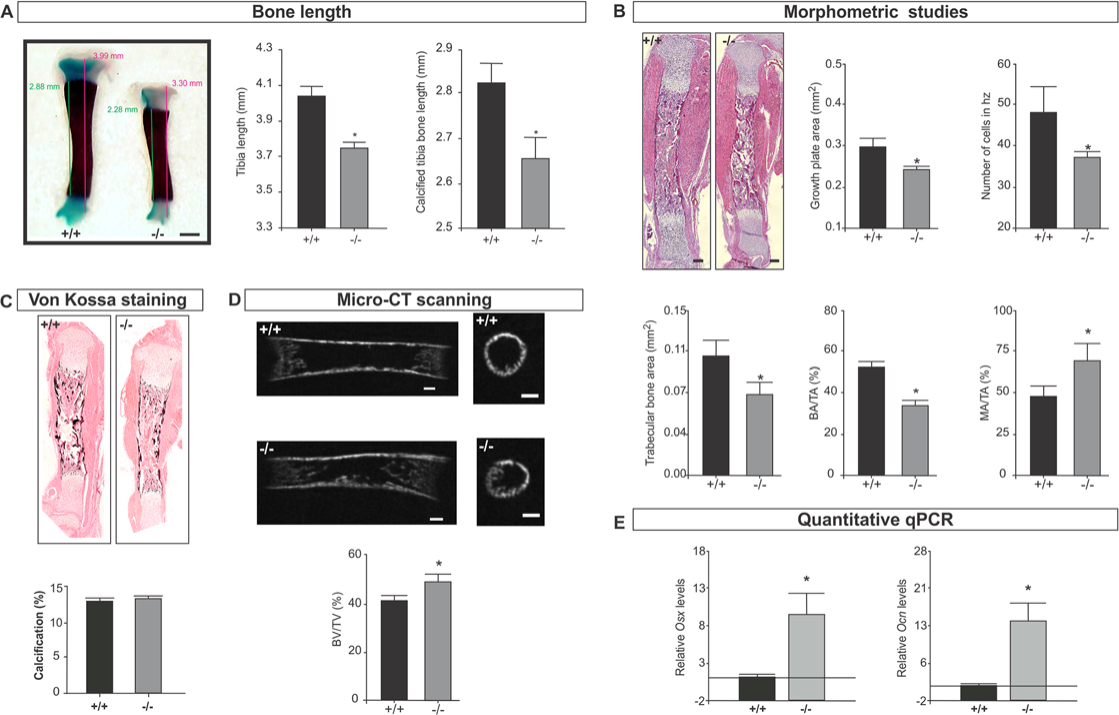
Tibia bone development is affected in *Spag17*-deficient mice. (A) Alcian Blue/ Alizarin Red staining on tibia from wild-type (+/+) and *Spag17*-mutant (-/-) mice. Measurement of tibia length and calcified tibia bone length shows *Spag17*-mutant limbs are shorter than wild-type mice. Scale bars, 0.5 mm. (B) Histological and morphometric studies on tibia shows cartilage and bone defects. Scale bars, 200 μm. (C) von Kossa staining. (D) Micro-CT structural analysis on tibias; mid-sagittal sections (left) and cross-sectional trans-axial views at the mid-diaphysis (right). Images were acquired in 600 projections in 180 degrees of rotation with 500 miliseconds exposure per projection. Scale bars, 200 μm. (E) mRNA expression of *osterix* (Osx) and *osteocalcin* (Ocn) measured by qPCR. (+/+), wild-type; (-/-), *Spag17*-mutant mice. Data are presented as means ± SEM from ≥7 mice. * Indicates statistically significant differences, p< 0.05. hz: hypertrophic zone; BA/TA: bone area/total area; MA/TA: marrow area/total area; BV/TV: bone volume/total volume; *Osx*: *osterix*; *Ocn*: *osteocalcin*.

Measurement of tibia and calcified bone length revealed a shorter distal limb on *Spag17*-mutant mice (Fig 2A). Morphometric studies showed reduced growth plate area, characterized by a decrease in the number of hypertrophic cells (terminally differentiated chondrocytes) (Fig 2B and S2 Fig). Also bone morphology was affected by *Spag17* disruption. Tibias had significantly less trabecular bone area, a reduction in the relation of bone area/total area (BA/TA%) and an increased ratio of marrow area/total area (MA/TA%) (Fig 2B). Calcification evaluation from von Kossa staining did not show statistically significant differences between wild-type and *Spag17*-mutant mice (Fig 2C and S2 Fig). However, micro-CT detected increased bone volume/total volume (BV/TV%) (Fig 2D). In addition, expression of *Ocn* and *Osx* was higher in the mutant mice in comparison to wild-type mice (Fig 2E). No statistically significant differences in the osteoclast number were observed in tibias comparing wild-type and knockout mice, S3 Fig These data suggest that the effect of *Spag17* in hind limb development depends on the maturation state of the individual bones. Reduced *Spag17* results in delayed tibial development. The shorter growth plate and the presence of mainly primary bone might explain the shorter bone length compared to wild type animals.

#### *Spag17*-deficient mice present fused sternebrae

**Fig 3:**
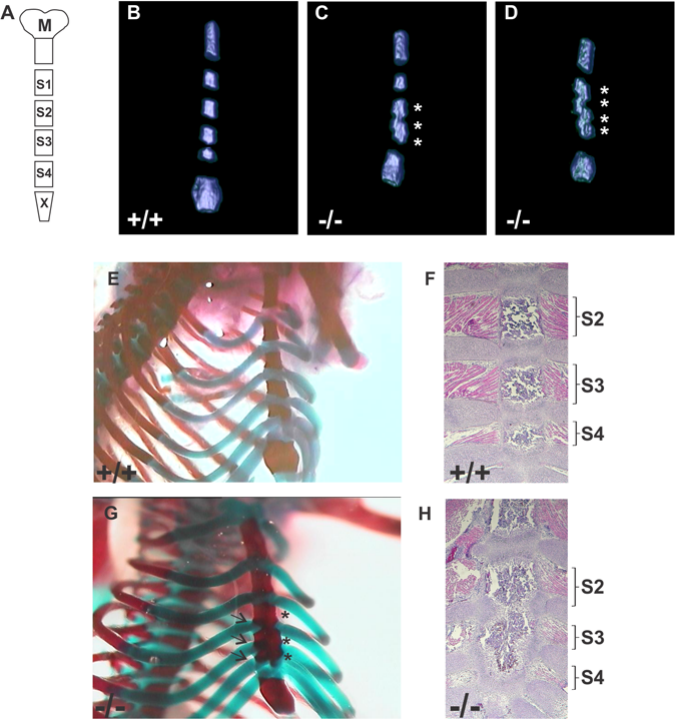
Disrupted development of the sternum in *Spag17* ^*-/-*^ mice. (A) Schematic representation of normal sternal bones (M, manubrium; S1 to S4, sternebrae 1 to 4; X, xiphoid). (B,C and D) Analysis of the sternum by micro-CT scanning shows fused sternebrae in the mutant mice. Pictures are representative images from respective supporting videos. (Fig 3B is representative of S1 Video; Fig 3C is representative of S2 Video; and Fig 3D is representative of S3 Video.) (E and G) Alcian blue/ Alizarin red staining. Failure of ribs 4, 5 and 6 to complete their attachment to the sternum and promote sternebrae separation is apparent in the stained preparations in the *Spag17* ^***-/-***^ mice (arrows). (F and H) Representative H&E staining shows the presence of normal cartilage development that prevents fusion of the vertebrae in wild type animals. (+/+), wild-type; (-/-), *Spag17*-mutant mice. * Fused sternebrae.

*Spag17* ^*-/-*^ mice had additional skeletal malformations. The sternum is a segmented structure (Fig 3A) consisting of the manubrium, four sternebrae, and a xiphoid [20]. Micro-CT showed *Spag17* ^*-/-*^ mice had fused sternebrae frequently (70%) between S3 and S4 and occasionally (20%) between S2, S3 and S4 (Fig 3C and S2 Video), but rarely (10%) between all sternebrae (Fig 3D and S3 Video). No fused sternebrae were observed in wild-type mice. Sternebrae malformations were also observed by Alcian blue/ Alizarin red staining (Fig 3G). The failure of ribs 4, 5 and 6 to complete their attachment to the sternum and promote sternebrae separation was apparent in the stained preparations in the *Spag17* ^*-/-*^ mice. Similarly, histological examination showed failure of the last ribs to complete their attachment to the sternum, leading fused sternebrae (Fig 3H).

#### Mineralization defects in skull and phalanges in *Spag17*-deficient mice

**Fig 4:**
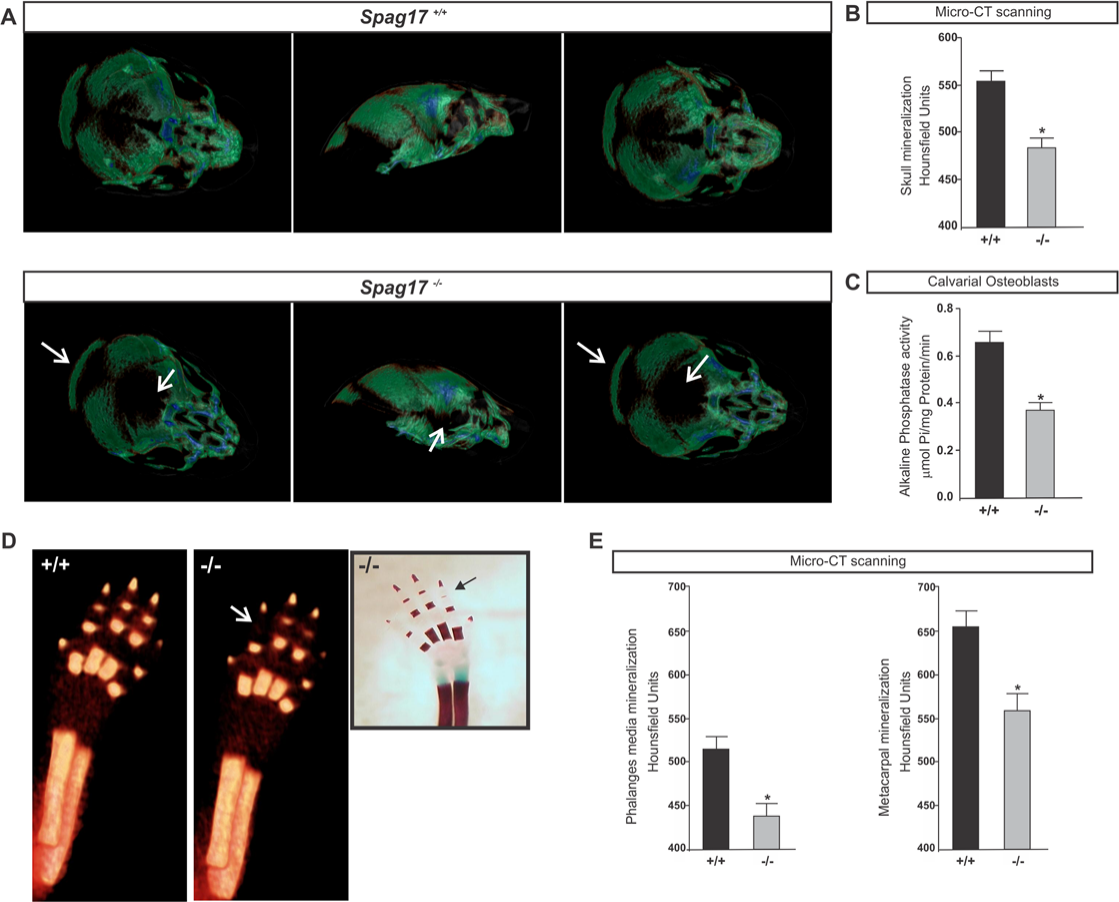
Cranial and phalanges defects in *Spag17* ^*-/-*^. (A) Micro-CT imaging of mineralized skull. Note the demineralization of the skull in the mutant mouse (white arrows). (B and E) Quantitative mineralization measurement by micro-CT scanning. (C) Alkaline phosphatase activity in cultured calvarial osteoblast. (D) Micro-CT imaging of mineralized forelimb. Insert shows reduced metacarpal mineralization by Alcian Blue/ Alizarin Red staining. Micro-CT images were acquired in 360 projections by 360 degree rotation, with 680 millisecond exposure per projection. White and black arrows indicate less metacarpal mineralization in the mutant mouse. (+/+), wild-type; (-/-), *Spag17*-mutant mice. Data are presented as means ± SEM. * Indicates statistically significant differences, p< 0.05.

Micro-CT studies in *Spag17*-mutants showed separation in the bone from skull, which may be related to the prominent hydrocephalous and the intracranial pressure (Fig 4A) [19]. In addition, there was reduced mineralization in the skull (Fig 4B), suggesting membranous ossification defects. Medial and metacarpal phalanges in *Spag17* ^*-/-*^ mice also showed reduced mineralization (Fig 4D and 4E). To investigate defects in mineralization, osteoblasts were isolated from calvaria and cultured using a standard protocol (see Materials and Methods), and alkaline phosphatase (ALP) activity, an early marker of osteoblast activity, was measured. Fig 4C shows a reduction in ALP activity in *Spag17*-mutant mice.

### Primary cilia are disrupted in *Spag17*-mutant mice

Cilia play important roles in proliferation, differentiation and functions of many cells. Ciliary defects also results in skeletal development abnormalities [21]. *Spag17* is expressed in tissues with “9+2” motile cilia like testis, brain, oviduct, lung and trachea [14,19]. However, *Spag17* expression in cartilage and bone has not been reported in mice. RT-PCR analysis on RNA isolated from articular cartilage and bone showed that this gene is indeed expressed in wild-type mice, S4 Fig

**Fig 5:**
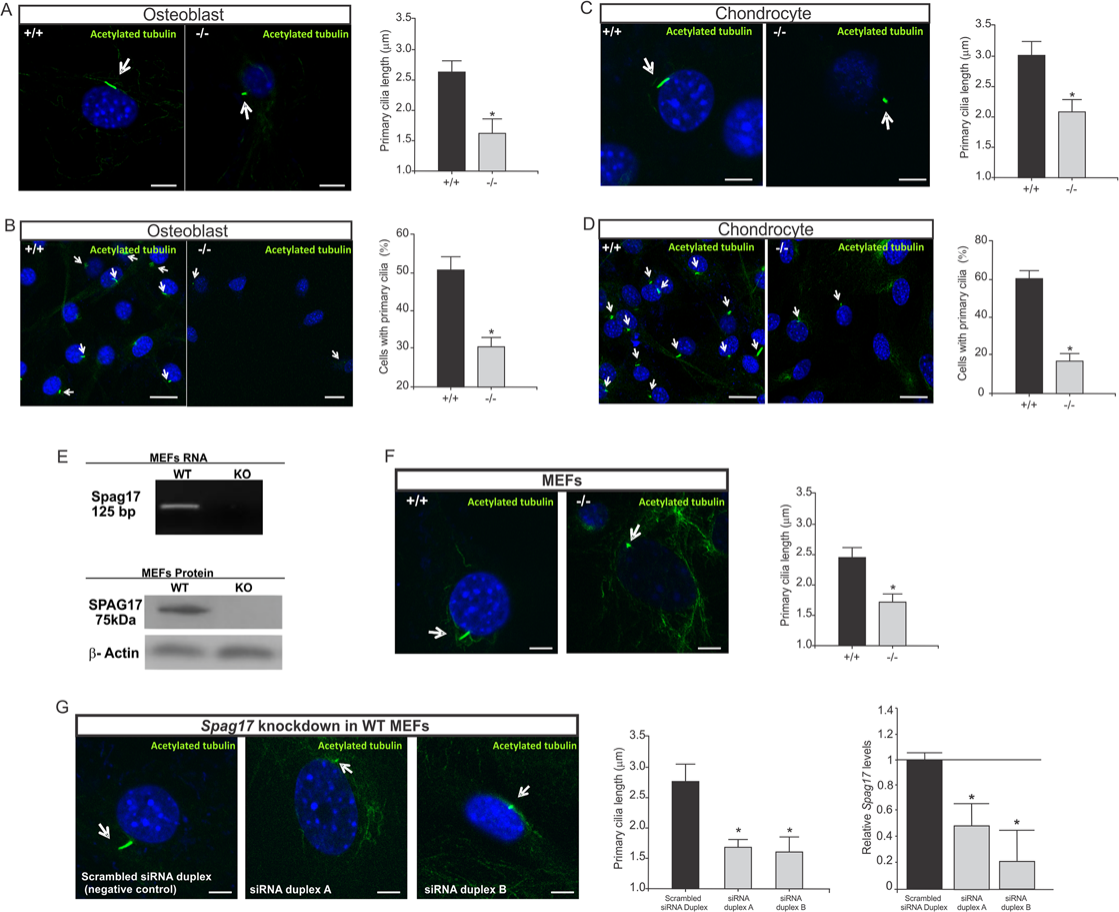
Primary cilia are altered in *Spag17*-deficient osteoblast, chondrocyte and MEFs cells. Cells were stained with anti-acetylated α-tubulin to visualize primary cilia and DAPI as a nuclei marker. (A) Primary cilia length from osteoblast cells. Scale bars, 5 μm. (B) Percentage of osteoblast cells expressing primary cilia. Scale bars, 20 μm (C) Primary cilia length from chondrocyte cells. Scale bars, 5 μm. (D) Percentage of chondrocyte cells expressing primary cilia. Scale bars, 20 μm. Mouse embryonic fibroblasts (MEFs) were isolated from embryos at E12.5. (E) Detection of *Spag17* mRNA and protein in MEFs. RT-PCR products were generated by primers from exon 4 and 5. The knockout mice have a deletion of the entire exon 5. Detection of SPAG17 protein by western blot using an antibody against C-terminal domain. (F) After serum-starvation to promote cilia growth, primary cilia were visualized in the MEFs with acetylated tubulin antibody, and DAPI as a nuclei marker. Primary cilia from *Spag17*-mutant MEFs were significantly shorter than wild-type. Scale bars, 5 μm. (G) *Spag17* knockdown in WT MEFs cells after treatment with *Spag17* siRNA duplex reproduced the shorter primary cilia phenotype. Scale bars, 5 μm. Arrows indicate primary cilia. (+/+), wild-type, (-/-); *Spag17*-mutant mice. Data are presented as means ± SEM. * Indicates statistically significant differences, p< 0.05.

In order to determine if primary cilia in bone and cartilage are affected in *Spag17*-mutant mice, cultures of osteoblasts that were isolated from calvaria and cultures of chondrocytes that were isolated from the costochondral cartilages from wild-type and *Spag17*-mutant mice were stained with anti-acetylated α-tubulin antibody (a primary cilia marker). Fig 5 shows that primary cilia from *Spag17* ^*-/-*^ mice are shorter than those from *Spag17* ^*+/+*^ mice, and there is a reduction in the percentage of cells expressing primary cilia in the knockout cells.

To further evaluate an effect of *Spag17* in primary cilia, mouse embryonic fibroblasts (MEFs) were isolated from wild-type and *Spag17*-mutant embryos at E12.5. Expression of SPAG17 in wild-type MEFs was shown by Western blot analysis (Fig 5E) using an anti-SPAG17 antibody against the C-terminal domain. This antibody detected a protein ~ 75 kDa molecular weight, suggesting that MEFs express a SPAG17 variant instead of the 250 kDa full length protein [14,18]. This ~75 kDa protein may be derived from alternative splicing or post-translational processing, processes [14]. SPAG17 mRNA was also detected in wild-type MEFs by RT-PCR (Fig 5E). After serum-starvation to promote cilia growth, primary cilia were visualized in the MEFs with anti-acetylated α-tubulin antibody. Primary cilia from *Spag17*-mutant MEFs were significantly shorter than wild-type (Fig 5F). The same primary cilia phenotype was reproduced by infecting wild-type MEFs with two different *Spag17* siRNA duplex molecules (Fig 5G).

## Discussion

The embryonic limb is first visible as a small bud that protrudes from the body and contains mesenchymal cells that are rounded by a layer of ectoderm. The limb skeleton is laid down progressively, starting from the proximal end to the distal end. The mesenchymal cells condense, differentiate into cartilage, and later into bone. Many genes are known to regulate growth and patterning of the limb in three dimensions; however, is not to clear how these genes interact [22]. The results showed here revealed an unexpected function of *Spag17* gene in skeletal development. It appears that mutation in *Spag17* differentially altered femurs and tibias. Femurs had mature Haversian bone formation, potentially leading to bone shortening due to premature ossification. Tibia bone shortening may be due to a delay in cartilage and primary bone development. These results suggest defects in limb patterning and also chondrocyte and osteoblast differentiation and maturation.

It is not clear yet how SPAG17 influences bone development and mineralization, and how its mutation can reduce and enhance this process in different bone locations as observed in skull and phalanges and limbs from mutant mice, respectively. The site-specific differences in the bone defects in *Spag17* ^*-/-*^ mice may be related to a location-specific cell signaling process. Whether these are a consequence of inherent differences in development or are the cause of differences in development is not clear. We have observed differential effects of gene deficiency in the femur and tibia in other mice models due to the rate at which different bones in the embryo develop [23]. The phenotypic changes in the femur of the *Spag17* ^*-/-*^ mice suggest increased bone formation. In contrast, changes in the tibia, which matures at a later time than the femur, indicate that the gene defect altered features of the growth plate. Similar developmentally related changes were observed in sternum, where mineral was observed in the intersternebral spaces at early times, when the presence of cartilage prevents fusion of the vertebrae in wild type animals. The observation that osteoclast number was not affected by *Spag17* knockout may suggest that the effect of *Spag17* in bone and cartilage was on the anabolic phase (bone formation and differentiation) and not on the remodeling phase. The effect of *Spag17* on bone development differentiation and calcification depends on the stage of bone maturation and the type of bone. One potential reason why calvaria and long bones respond differentially to the lack of this important cilia protein are their distinct functions, which lead to differences in the mechanical forces they undergo and to which the cilia are exposed.

Normal sternum development requires that all the ribs form a continuous bar of cartilage between sternebrae by embryonic day 16 [20], suggesting that the defects in the *Spag17* ^*-/-*^ mice may be due to alterations in early skeletal development. *Fgfr1-*mutant mice develop a similar fused sternum phenotype as observed in *Spag17* ^*-/-*^ mice [24]. Additionally, *Runx1* has been shown to play an essential role in the development of the sternum [25], and *Runx2* cooperates with *Runx1* during sternal morphogenesis [26]. However, the link between these genes and their possible regulation by *Spag17* remains to be clarified.

Mechanotransduction is a process that helps bone cells to convert mechanical forces into biochemical signals. It is important for maintaining bone health and homeostasis [27]. Primary cilia extend from the surface of cells and function mainly as sensory antenna [28]. This organelle is believed to play a role in bone mechanotransduction [27]. In this context, calcium deposition by osteoblasts has been shown to be mediated by primary cilia [29]. Cultures of MLO-A5 cells (murine long osteocyte cell line A5), which display shorter and fewer cilia per cell, show reduced mineralization [29]. The same phenotype was observed in calvarial osteoblasts from *Spag17*-mutant mice suggesting this may be one of the mechanisms where *Spag17* plays a role and has an effect in bone mineralization. In addition, several cell signaling pathways take place in primary cilia development and function. For instances, vesicular trafficking, intraflagellar transport (IFT), Hedgehog (Hh) and Wnt signaling converge in the organization and functional maintenance of the primary cilium [30,31]. Similar pathways influence cartilage and bone development and growth [32]. Ciliary chondrodysplasias are believed to result from imbalances in the signaling pathways that normally occur in functional cilia. Moreover, several knockout mouse models for genes related to primary cilia made important contribution to the knowledge of skeletal development [33].

Our understanding of how disruption of the *Spag17* gene leads to alterations in primary cilia function and subsequent cellular pathology is limited. It is unknown, if this CPC protein is related to a structural function, as primary cilium lack the CPC, or it may plays a role in any of the signaling (vesicular trafficking, intraflagellar transport (IFT), Hedgehog (Hh), Wnt signaling, etc). Our results show that primary cilia are shorter in knockout mice. We hypothesize that *Spag17* may be involved in ciliogenesis like *Spag6* [34], although its role and/or mechanisms related to ciliogenesis may be different from *Spag6*. In addition, a ~75 kDa SPAG17 protein is expressed in MEFs suggesting that it is a variant instead of the 250 kDa full length protein. This ~75 kDa protein may be derived from alternative splicing or post-translational processing, processes. Further studies are required to determine how this protein functions in primary cilia.

Several studies have reported an association of *SPAG17* gene variants with height [1,7,9–13]. However, until now there has been no known link between this gene, whose function was thought to be restricted to motile cilia, and height. Our findings have revealed functions for *Spag17* that extend the role of this gene to regulation of skeletal differentiation, growth and mineralization, perhaps because of a function in primary cilia. This newly defined role could explain the influence of SPAG17 on linear growth and bone development.

## Materials and Methods

### Mice

All animal procedures were performed in accordance with the National Research Council’s Guide for the Care and Use of Laboratory Animals. Animals were euthanized in accordance with protocol AD10000167 approved by the Virginia Commonwealth University Institutional Animal Care and Use Committee. Target disruption of *Spag17* was made by deletion of exon 5 using Cre-lox and FLP-FRT recombination system. Briefly, embryonic stem (ES) cells were obtained from the KOMP Repository (Davis, CA). The chimera males were crossed to C57BL/6J wild-type females, the resulting heterozygous offspring were crossed to the 129S4/SvJaeSor-Gt(ROSA)26Sor^tm1(FLP1)Dym^/J to remove the Neo cassett*e*. The *Spag17* ^*+/flox*^ mice were used for mating with *CMV-Cre* (B6.C-Tg(CMV-cre)1Cgn/J) mice to generate *Spag17* ^*+/-*^ *;CMV-Cre* mice. Male and female *Spag17* ^*+/-*^ *;CMV-Cre* mice were mated resulting in the expecting Mendelian inheritance for *Spag17* ^*-/-*^ *;CMV-Cre* mice. After these mattings the mice had a mixed background of C57BL/6J−129S4/SvJ. Deletion of this gene was published in detail in Teves et al., 2013 [19].

### Histology studies

Bones were removed from newborn mice and fixed overnight in 4% formalin/PBS, dehydrated in ethanol and embedded in paraffin. Slides were stained with hematoxylin and eosin. Histology preparations were performed at the VCU Massey Cancer Center Macromolecule Core Facility, Virginia Commonwealth University.

### Morphometric studies

Histological sections were imaged (Zeiss AxioObserver, Germany) using a 20x objective. Cartilage (growth plate area, percent hypertrophic cells in the growth plate, percent resting zone cells, height of hypertrophic region, number of cells in hypertrophic region) and bone (total bone area, cortical bone area, trabecular bone area, bone area/total area, and marrow area/total area) were measured in the tibia and femur using Zen Black software (Zeiss). Data presented are mean ± standard error of n = 7 mice. Osteoclast number was counted in 8 knockout and Wild-type mice. Statistical comparisons were made using unpaired t-test; p < 0.05 was considered significant.

### High resolution micro-CT

Newly born mouse pups were euthanized using isoflurane, and the hindlimb and sternum were removed and fixed in 10% neutral buffered formalin. High resolution microcomputed tomography (micro-CT) was performed using the Brϋker Skyscan 1173 micro-CT scanner (Skyscan NV, Kontich, Belgium). Sternal and hind limb images were acquired in 600 projections in 180 degrees of rotation, with 500 millisecond exposure per projection, at 50 kilovolts and 160 microamps, an image pixel size of 7.91 micrometers, and a rotation step of 0.40 degrees. Images were acquired at a resolution of 2240 x 2240 pixels. Sample images were reconstructed standard Feldkamp reconstruction using NRecon Software (Kontich, Belgium) and a Gaussian smoothing kernel of 2. Region of Interest (ROI) analysis was conducted using CT-analysis software (CTAn, version 1.13.11.0, Skyscan NV) to assess the amount of attenuated tissue present as a percentage of the total three dimensional volume in the ROI (% Bone Volume/Total Volume, %BV/TV). Data are mean ± standard error of n = 7 mice and statistical comparisons were made using unpaired t-test; p < 0.05 was considered significant.

### Measurement of bone length and width

Alcian blue/ Alizarin red stained hind limbs were scanned with Life Technologies EVOS Fl Auto Cell Imagining System. Then images were analyzed, and bone measured by ImageJ software (NIH). Data are mean ± standard error of n = 9 mice and statistical comparisons were made using t-test; p < 0.05 was considered significant.

### RT-PCR and qPCR

Total RNA was isolated with TRIzol (Life Technologies, Inc., Grand Island, N.Y.) from newborn mice sternum, cartilage, and bone tissue. The RNA was reversed transcribed with RETROscript kit (Ambion, Austin, TX) according to the manufacturer’s instructions, and the cDNA were used for RT-PCR or qPCR. Amplification products were resolved on 1% agarose gels stained with ethidium bromide. The results presented are representative of seven different animals from each genotype shown.

### Micro-CT scanning

Mouse pups were euthanized with excess of isoflurane and were then imaged in the micro-CT scanner. Micro-CT was performed using an Inveon micro-CT system (Siemens, USA). Three dimensional images were acquired in 360 projections by 360 degree rotation, with 680 millisecond exposure per projection, around a 31 x 31 mm field of view (FOV), with 75 kV voltage, 500 μA of current and 2 × 2 binning were used to yield an effective pixel size of 19.24 μm and a scan time of 6 minutes. A thin walled plastic tube containing water was scanned using the same parameters and was used for Hounsfield unit (HU) normalization. HU normalized micro-CT images were reconstructed using a modified Feldkamp algorithm using manufacturer provided software (COBRA Exiim). ROI analyses for bone density and bone length measurements were done in the Inveon Research Workplace 4.1 (Siemens Healthcare, PA). Statistical comparisons were made using Student’s t-test; p < 0.05 was considered significant.

### Alcian blue/ Alizarin red staining

Skeletons from newborn mice were stained with Alcian Blue/ Alizarin Red as described by Ovchinnikov D [35]. Skeletal preparations were analyzed and photographed in glycerol/ethanol solution.

### von Kossa staining

Bones were removed from newborn mice and fixed overnight in 4% formalin/PBS, dehydrated in ethanol and embedded in paraffin. Slides were stained for phosphate via the method of von Kossa at the VCU Massey Cancer Center Macromolecule Core Facility, Virginia Commonwealth University using a kit and following manufacturer’s instructions (American MasterTech Scientific Inc., CA, USA).

### Osteoblast and chondrocyte isolation and culture

Newborn mice were euthanized by an overdose of isoflurane. Chondrocytes were isolated from the costochondral cartilage following methods previously described for chondrocyte isolation for rats [36]. Briefly, rib cages were removed and resting zone cartilage dissected. Cartilage pieces were washed in Hank's Balanced Salt Solution (HBSS), incubated in 0.25% trypsin-EDTA (Life Technologies) for 15 minutes, then in 0.02% collagenase type II (Worthington Biochemicals, Freehold, NJ) for 3 hours. Cells were separated from tissue debris using 40 μm cell strainers (BD, Franklin Lakes, NJ) and collected by centrifugation. Cells were cultured in DMEM supplemented with 10% fetal bovine serum (FBS, Life Technologies), 1% penicillin-streptomycin, and 50 ug/ml ascorbic acid (Sigma Aldrich). Osteoblasts were isolated from frontal and parietal bones as previously described [37]. Briefly, minced bone pieces were digested in trypsin followed by 0.01% collagenase type I (Sigma Aldrich) and 0.01% dispase (Life Technologies). Cells were separated from debris using cell strainers and collected by centrifugation. Cells were cultured in DMEM supplemented with 10% FBS and 1% penicillin-streptomycin. Passage one chondrocytes and osteoblasts were used for subsequent experiments.

### MEFs isolation and culture

Mouse embryonic fibroblasts were isolated from E12.5 embryos. Head and heart tissues were removed, and the remaining embryos were digested with 0.25% trypsin/EDTA at 37°C for 15 min. After digestion and removal of undigested tissues, the cells were plated into a 100 mm dish, and allowed to grow in DMEM + 10% FBS. Early passages cells were used for all the experiments. *Spag17* was knocked down in WT MEFs by treatment with *Spag17* (mouse) -3 unique 27mer siRNA duplex (OriGene Technologies, Inc. Rockville, MD).

### Alkaline phosphatase activity

The assay was performed following standard techniques. Briefly, confluent cultures of mouse osteoblasts were lysed in 0.05% Triton X-100 (Sigma Aldrich) and homogenized by sonication for 10 seconds. Alkaline phosphatase specific activity was measured in the cell lysates as the release of *p*-nitrophenol from *p*-nitrophenylphosphate at pH 10.2. Enzyme activity was normalized to total protein content of the lysates (BCA Assay, ThermoFisher).

### Immunofluorescence studies

Osteoblasts and chondrocytes in passage one were fixed in 4% formalin for 1 hour at room temperature and blocked with 10% goat serum diluted in PBS containing 3% BSA and 0.2% Triton X-100. Then, cells were incubated with anti-acetylated α-tubulin mouse antibody overnight at 4°C. After several washes in PBS, cells were stained with anti-mouse IgG alexa-488 labeled antibody (dilution 1: 3,000). Finally, slides were sealed using VectaMount with DAPI (Vector Laboratories, Burlingame, CA). Images were taken by confocal laser-scanning microscopy (Leica TCS-SP2 AOBS). The same immunofluorescence staining protocol was used for MEFs cells. The results presented are representative of mean ± standard error obtained from four different animals of each genotype. Statistical comparisons were made using t-test; p < 0.05 was considered significant.

### Western Blot

Equal amounts of protein (100 μg/lane) were heated to 95°C for 10 min in sample buffer, loaded onto 12% SDS-PAGE gels, electrophoretically separated, and transferred to PVDF membranes (Millipore, Billerica, MA) by semi-dry transference. A dual-color precision plus protein standard (BIO-RAD, Hercules, CA) was used. Membranes were blocked for 1 h in 5% milk-TTBS (BIO-RAD, Hercules, CA) and then incubated overnight with Anti- SPAG17 C-terminal antibody (1:5,000) at 4°C, After several washes in TTBS, the membranes were incubated with an anti-rabbit IgG horseradish-peroxidase labeled antibody (1:2,000 dilution) for 1 h at room temperature. Protein was detected with Super Signal Chemiluminescent Substrate (Pierce). β-actin antibody was used as loading control to assure relatively equivalent amounts of protein were present amongst sample types.

### Statistical Analysis

InfoStat software was used for statistical analyses (Di Rienzo J.A., Casanoves F., Balzarini M.G., Gonzalez L., Tablada M., Robledo C.W. InfoStat ver 2009. Group InfoStat, FCA, Universidad Nacional de Córdoba, Argentina). Data are presented as means ± standard error. Normality of data was assessed by D'Agostino & Pearson omnibus normality test. Each set of data was analyzed comparing the means from two groups (wild-type and knockout) by Student’s t-test or one-way analysis of variance (ANOVA) followed by Duncan’s test for three group comparisons. Samples were considered significantly different when the p-value was < 0.05.

### Ethics statements

All animal procedures were performed in accordance with the National Research Council’s Guide for the Care and Use of Laboratory Animals. Experiments were in accordance with protocol AD10000167 approved by the Virginia Commonwealth University Institutional Animal Care and Use Committee.

## Supporting Information

S1 Fig Femur bone development is affected in *Spag17*-deficient mice. (A) Representative high magnification pictures from H&E staining on femur from wild-type (+/+) and knockout (-/-) mice. (B) Representative high magnification pictures from von Kossa staining on femur from wild-type (+/+) and knockout (-/-) mice.
(TIF)

S2 Fig Tibia bone development is affected in *Spag17*-deficient mice. (A) Representative high magnification pictures from H&E staining on tibia from wild-type (+/+) and knockout (-/-) mice. (B) Representative high magnification pictures from von Kossa staining on tibia from wild-type (+/+) and knockout (-/-) mice.
(TIFF)

S3 Fig Osteoclasts number is not statistically different in wild-type and Spag17-mutant mice. (A) Osteoclasts number in femur from wild-type (+/+) and knockout (-/-) mice. (B) Osteoclasts number in tibia from wild-type (+/+) and knockout (-/-) mice. Data are presented as means ± SEM from 8 mice for each group. No statistically significant differences were found between the two groups, p> 0.05.
(TIFF)

S4 Fig *Spag17* expression in the skeleton. RT-PCR products generated by primers from exon 4 and 5. Notice the knockout mice have a deletion in the entire exon 5 [19]. RNA was isolated from wild-type and *Spag17*-mutant sternum (S), limb bone (B) and cartilage (C).
(TIF)

S1 Video Analysis of the sternum by micro-CT scanning from *Spag17* ^*+/+*^ (+/+) mouse. (MOV)

S2 Video Analysis of the sternum by micro-CT scanning from *Spag17* ^*-/-*^ (-/-) mouse showing fusion of vertebrae S2, S3 and S4. (MOV)

S3 Video Analysis of the sternum by micro-CT scanning from *Spag17* ^*-/-*^ (-/-) mouse showing fusion of all vertebrae. (MOV)
