# Supplementary figures and images for: Spag17 Deficiency Results in Skeletal Malformations and Bone Abnormalities
Source: PLoS One. 2015 May 27;10(5):e0125936. doi: 10.1371/journal.pone.0125936 (PMC4446355; doi:10.1371/journal.pone.0125936)

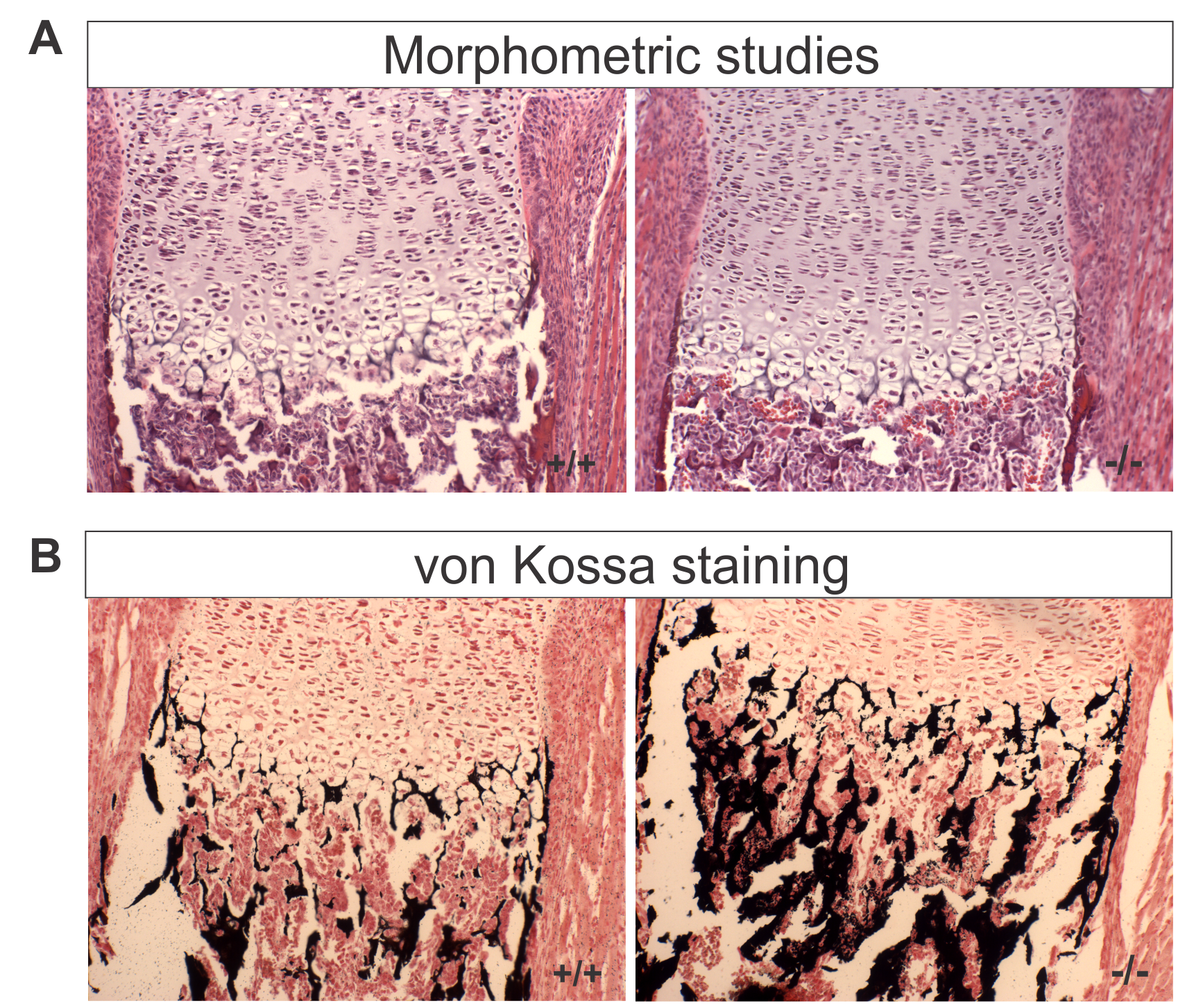

Supplement: S1 Fig — (A) Representative high magnification pictures from H&E staining on femur from wild-type (+/+) and knockout (-/-) mice. (B) Representative high magnification pictures from von Kossa staining on femur from wild-type (+/+) and knockout (-/-) mice. (TIF) [file pone.0125936.s001.tif]

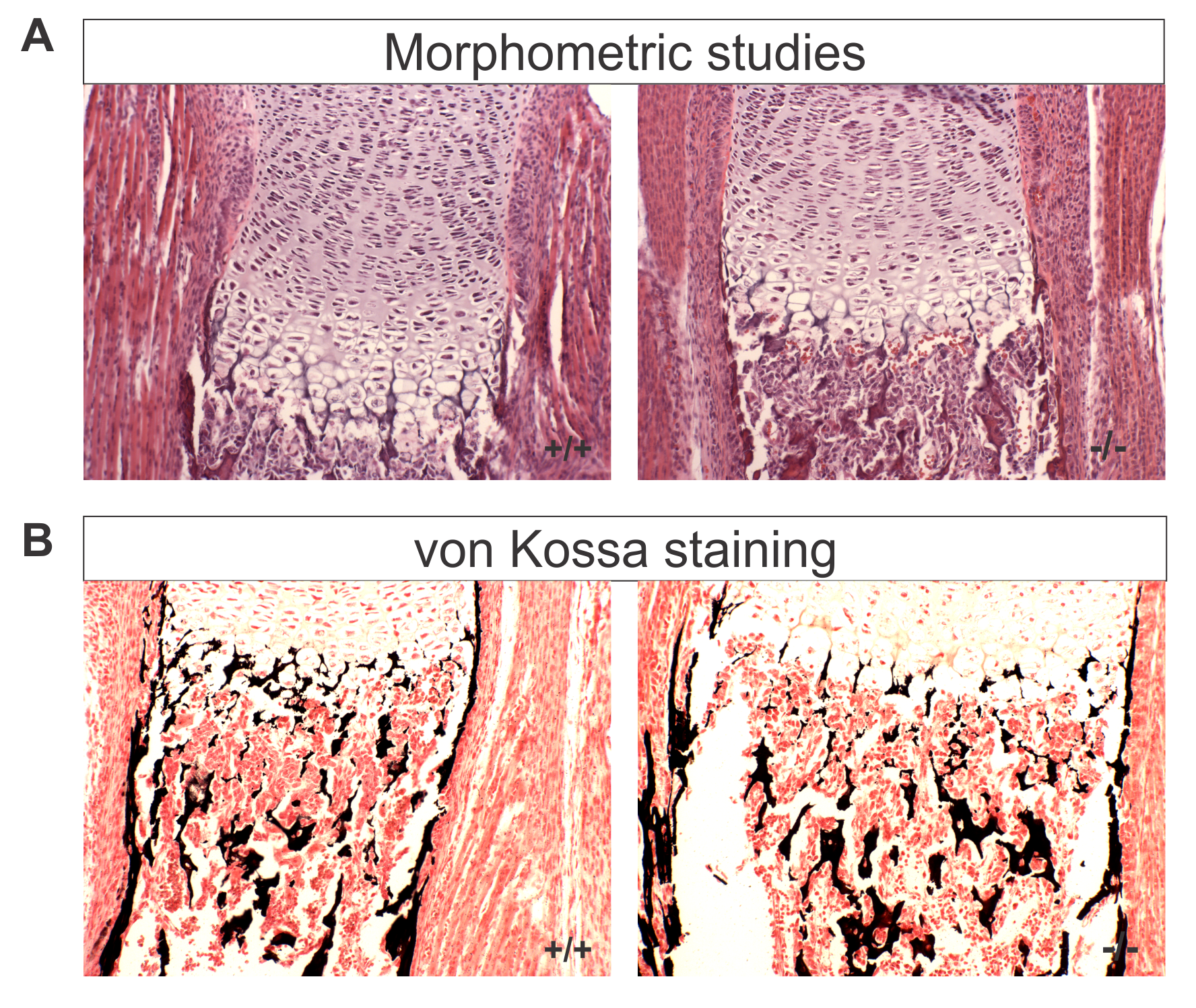

Supplement: S2 Fig — (A) Representative high magnification pictures from H&E staining on tibia from wild-type (+/+) and knockout (-/-) mice. (B) Representative high magnification pictures from von Kossa staining on tibia from wild-type (+/+) and knockout (-/-) mice. (TIFF) [file pone.0125936.s002.Tiff]

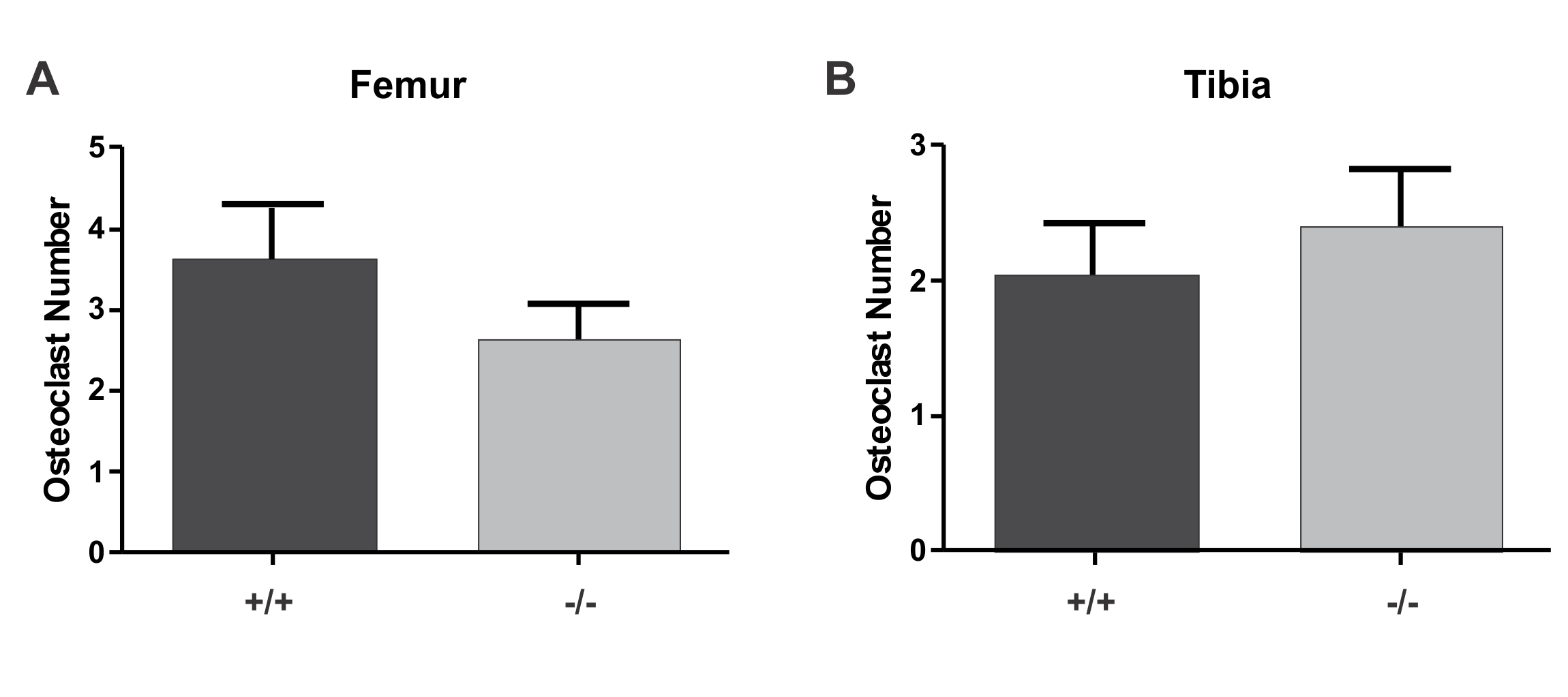

Supplement: S3 Fig — (A) Osteoclasts number in femur from wild-type (+/+) and knockout (-/-) mice. (B) Osteoclasts number in tibia from wild-type (+/+) and knockout (-/-) mice. Data are presented as means ± SEM from 8 mice for each group. No statistically significant differences were found between the two groups, p> 0.05. (TIFF) [file pone.0125936.s003.Tiff]

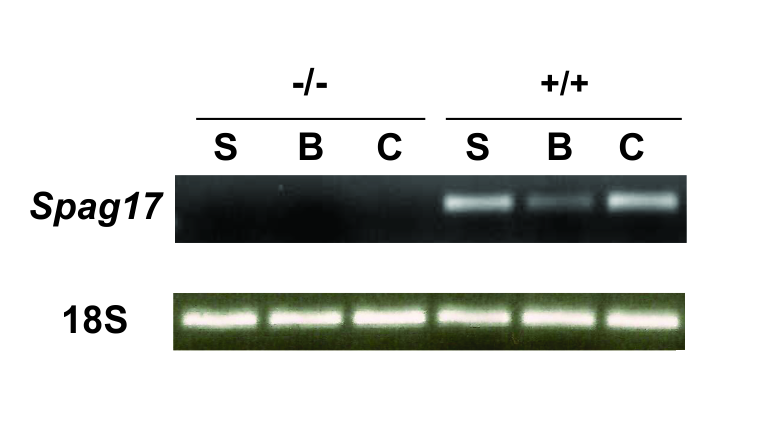

Supplement: S4 Fig — RT-PCR products generated by primers from exon 4 and 5. Notice the knockout mice have a deletion in the entire exon 5 [19]. RNA was isolated from wild-type and Spag17-mutant sternum (S), limb bone (B) and cartilage (C). (TIF) [file pone.0125936.s004.tif]
